# New Roles for Old Friends: Involvement of the Innate Immune System in Tumor Progression

**DOI:** 10.3390/ijms24087604

**Published:** 2023-04-20

**Authors:** María Castaño, Eva González-Cantó, Cristina Aghababyan, Sarai Tomás-Pérez, Julia Oto, Raquel Herranz, Pilar Medina, Martin Götte, Bárbara Andrea Mc Cormack, Josep Marí-Alexandre, Juan Gilabert-Estellés

**Affiliations:** 1 Haemostasis, Thrombosis, Arteriosclerosis and Vascular Biology Research Group, Medical Research Institute Hospital La Fe, 46026 Valencia, Spain; 2 Research Laboratory in Biomarkers in Reproduction, Gynaecology, and Obstetrics, Research Foundation of the General University Hospital of Valencia, 46014 Valencia, Spain; 3 Department of Obstetrics and Gynecology, General University Hospital of Valencia Consortium, 46014 Valencia, Spain; 4 Department of Gynecology and Obstetrics, Münster University Hospital, 48149 Muenster, Germany; 5 Department of Pathology, General University Hospital of Valencia Consortium, 46014 Valencia, Spain; 6 Department of Pediatrics, Obstetrics, and Gynecology, University of Valencia, 46014 Valencia, Spain

The association between the immune system and tumor progression has attracted much interest in the research community in recent years. The immune system, constituted by varied cell types that perform a coordinated response, is classified into innate and adaptive immunity. Effectors of innate immunity include neutrophils, macrophages, monocytes, eosinophils, basophils, and natural killer cells (NK). On the other hand, adaptive immunity involves T and B lymphocytes, which present a specific and structurally unique receptor [1].

The adaptive immune system has been the focus of research attention, which led to a better understanding of how lymphocytes interact with tumor cells, the discovery of immune checkpoints, and the development of targeted therapies. Initially, most efforts to identify and select antigens for therapeutic targeting cancer focused on shared tumor antigens, as they can be used to treat a wide range of cancer patients. However, it is becoming increasingly clear that many of these shared antigens are also expressed by self-tissues, either in peripheral cells or the thymus, which can lead to immunologic tolerance for the highest avidity interactions between peptide, major histocompatibility complex I and T-cell antigen receptor. As a result, immune responses against these antigens can be restricted to lower-affinity interactions, which may limit therapeutic efficacy [2].

Moreover, immune checkpoint inhibitors (ICIs) have become the first-line therapy for various solid and liquid tumor types. ICIs act by releasing the inhibitory brakes of T-cells, leading to strong activation of the immune system and effective antitumor immune responses. Currently, the most commonly used immunotherapeutic agents are blocking antibodies that target immune inhibitory receptors, such as CTLA-4 and PD-1 [3]. For instance, three anti-PD-L1 antibodies (atezolizumab, durvalumab, and avelumab) are primarily used to treat urothelial carcinoma, non-small cell lung cancer, Merkel cell carcinoma, and melanoma, among others [3,4]. Nevertheless, while many patients experience a significant reduction in tumor size as a result of ICIs, others with different tumor types do not respond to these therapies, as occurs for metastatic breast cancer [5] and ovarian cancer, in which clinical trials have reported poor outcomes in terms of patient response and survival [6]. Among patients who initially respond, some eventually develop resistance and experience tumor relapse, which is known as acquired resistance [3]. Chimeric antigen receptor (CAR) T-cells are also extensively studied as potential adjuvant therapies for cancer treatment. These cells are designed to promote specific T-cell responses and can be used in combination with surgical resection and chemotherapy to treat various types of cancer [7]. CAR T-cell treatment has provided remarkable results in hematological tumors but not in solid tumors, where T-cells encounter substantial difficulties in penetrating and surviving in the tumor microenvironment (TME) [8].

Undoubtedly, the involvement of the innate immune system in human cancer development and progression is now receiving increasing recognition. Thereby, the identification of various elements of the innate immune system such as tumor-associated macrophages (TAMs) and neutrophils (TANs), myeloid-derived suppressor cells (MDSCs), and NK cells in the TME has motivated researchers to investigate its role in creating a favorable environment for tumor growth and metastasis. Here we intend to briefly summarize the involvement and relevance of each of them.

Neutrophils, the most abundant leukocytes in humans, have diverse and critical roles in immunity. Traditionally, they have been considered part of the innate immune system constituting the first line of defense at sites of infection and injury [9]. Surprisingly, high levels of TANs have been linked with an adverse prognosis in different malignancies such as renal cell carcinoma, melanoma, colorectal cancer, hepatocellular carcinoma, glioblastoma, gastric, esophageal, lung, ovarian, and neck cancer [9]. Similarly to the M1/M2 phenotype of macrophages, two polarization states of TANs have been proposed, termed N1 and N2, representing anti-tumor and pro-tumor populations, respectively [10]. Moreover, in response to different stimuli, neutrophils have proven to be capable of releasing NETs composed of DNA, citH3, and cytoplasmic and granular proteins (such as calprotectin, MPO, and elastase) in a process called NETosis [11]. In cancer, those NETs are reportedly able to awaken dormant cancer cells, promoting cancer relapse and metastasis. In addition, they can be involved in inhibiting the immune response through evasion of adaptive immunity and ICIs, resistance to oncologic therapies, and entraping of circulating cancer cells, enhancing metastasis spread [12]. Hence, targeting NETs could represent a promising tool in cancer treatment since their markers in biofluids may serve as predictive biomarkers and their 3D structures as therapeutic targets. Interestingly, NETosis markers have also been shown to be useful indicators of other cancer-related processes, such as thrombotic complications. CitH3 has been presented as a predictor of venous thromboembolic events in cancer patients [13]. Additionally, in biliopancreatic cancer patients, calprotectin measured at diagnosis has been proposed as a biomarker to predict future venous thromboembolic events during follow-up [14]. In glioma, cell-free DNA (cfDNA) and MPO levels in plasma measured before surgery have also been proposed as predictors of incidental post-surgical pulmonary embolism in these patients [15]. Finally, plasma cfDNA and calprotectin have been postulated as potential minimally-invasive biomarkers of high-grade serous ovarian cancer that may improve current diagnostic markers [16].

Macrophages are innate immune cells that differentiate from circulating monocytes after extravasation into tissues. In oncological patients and preclinical experimental models, TAMs correlate with poor prognosis and reduced overall survival [10]. TAMs have two functionally polarized phenotypes in the TME: classically activated M1 and alternatively activated M2 macrophages. In the initial stages of various tumors, TAMs are preferentially polarized toward the M1 phenotype exerting anti-tumor functions. However, during cancer progression, changes in the TME promote the gradual shift towards a polarized M2 phenotype and exhibit distinct pro-tumorigenic functional properties [7,10,17]. Furthermore, monocytes and macrophages can extrude extracellular traps (MoETs and METs, respectively) that are composed of nuclear DNA fibers with myeloperoxidase (MPO), citrullinated histoneH3 (citH3), matrix metalloproteinase 9 and 12, and lysozyme [18]. Recently, a study from Xu et al. revealed that METs participate in tumor progression and identified several sources of NETs and METs in tumor tissues isolated from patients with pancreatic neuroendocrine cancer. The authors concluded that these extracellular traps can be used as biological indicators of patient prognosis [19]. Since the presence of MoETs and METs correlates with a worse prognostic in cancer patients, they could be useful as novel diagnostic and prognostic biomarkers.

MDSC are generated in the bone marrow and rapidly differentiate into macrophages, dendritic cells, neutrophils, eosinophils, basophils, and mast cells in healthy individuals. Thus, MDSCs are a heterogeneous collection of activated immature myeloid cells, comprising a mixture of granulocytic and monocytic subtypes [9]. They can be both polymorphonuclear (PMN-MDSCs) or mononuclear (M-MDSCs). PMN-MDSCs are morphologically and phenotypically similar to neutrophils while M-MDSCs are similar to monocytes [7]. During cancer, MDSCs can migrate to peripheral lymphoid tissues and tumor sites, contributing to TME formation [9]. Consequently, MDSCs have recently emerged as primarily responsible for the immune system blockade observed in cancer [20], since they are present in most cancer patients and have been shown to inhibit cytotoxic T-cell function and block T-cell enrichment in the tumor [9]. Since PMN-MDSCs are generally short-living cells, the differentiation has been studied in more detail for M-MDSCs into TAMs. Accordingly, M-MDSCs represent a potential therapeutic target for cancer therapy, not only because of their ability to suppress immune responses but also because of their high plasticity and differentiation potential. Therefore, it would be interesting that therapeutic targeting includes the blockade of M-MDSC migration to the tumors, the inhibition of M-MDSC differentiation into TAMs, and TAMs polarization [20].

Finally, NK cells are innate immune cells that display rapid and potent cytolytic activity in response to infected or transformed cells and possess a well-documented anti-tumor effect. The presence of NK cell infiltration in some types of cancers, such as colorectal and gastric, has been correlated with a favorable outcome [10]. Therefore, as a significant player in the innate immune response, NK cells present remarkable potential in clinical practice. In recent years, research into NK cell immunotherapy has experienced a boost. The latest developments mainly focused on cytokine supplements, monoclonal antibodies, modification of internal signal pathways, and genetic engineering of NK cells. Moreover, NK-cell-based therapy has achieved favorable results used either alone or in combination with other therapies, suggesting a wide and effective use in malignancies. Some of the therapies designed for NK cells are under clinical trials, such as monoclonal antibodies targeting the NK immunoglobulin-like receptor, anti-KIR (IPH2101, lirilumab), and those targeting ICI of NK, anti-NKG2A (monalizumab) [21].

Even though the adaptive immune system contribution has received considerable attention for several years in cancer, the involvement of the innate immune system in human oncology is now receiving increasing recognition. The identification of various innate immune system effectors in the TME has prompted researchers to investigate their role in tumor progression and metastasis, leading to the development of potential targeted therapies. Undoubtedly, this field is in constant development and much remains to be unraveled. Therefore, we encourage researchers to share their latest findings in this promising field related to the management of oncologic disorders.
